# Opportunities and challenges of a population health management approach for improving young people’s mental health

**DOI:** 10.1093/eurpub/ckaf200

**Published:** 2025-11-04

**Authors:** Jane Wills, Jessica T Oha, Gemma Bridge, Patrick Callaghan, Chris Flood, Catherine Jenkins, Paula Reavey, Susie Sykes

**Affiliations:** College of Health and Life Sciences, London South Bank University, London, United Kingdom; College of Health and Life Sciences, London South Bank University, London, United Kingdom; College of Health and Life Sciences, London South Bank University, London, United Kingdom; College of Health and Life Sciences, London South Bank University, London, United Kingdom; College of Health and Life Sciences, London South Bank University, London, United Kingdom; The Academy, Central London Community Health Care Trust, London, United Kingdom; College of Health and Life Sciences, London South Bank University, London, United Kingdom; College of Health and Life Sciences, London South Bank University, London, United Kingdom; College of Health and Life Sciences, London South Bank University, London, United Kingdom

## Abstract

Population Health Management uses available data to tailor services to identified and latent needs. It is advocated by the integrated care system in England, yet challenges remain regarding data availability, linkage, and application. This paper reports on the adoption of a population health management approach to design a complex programme aimed at improving young people’s mental health. In-depth qualitative interviews were conducted with local government public health professionals (PH) (*n = *5), intervention leads (*n = *3), and one focus group of young contributors to intervention design (*n = *5) to explore how population health management informed programme design and was perceived by stakeholders. Data were analysed using Delve.io. Key learning for public health included: (i) Data analysis for the PHM approach was strengthened by a dedicated data scientist, though some regarded the PHM approach as not new; (ii) Routine data had limited capacity to fully identify need, and linking datasets across health, social care, and education remained difficult; (iii) Local insights and co-production with young people were critical in identifying target groups not visible in routine datasets. Routine health data capture only part of the picture, often reflecting those already in contact with services. PHM approaches in public health need to integrate qualitative insights and local intelligence alongside quantitative analysis to address inequalities effectively.

## Background

Although Population Health Management (PHM) has been widely adopted to integrate healthcare organizations [1], its application in public health is relatively recent. PHM is a systematic means of proactively addressing population health by identifying those at risk and tailoring services to groups with shared needs [2]. It moves beyond clinical indicators to incorporate social determinants of health, using data from sectors such as education, housing, and criminal justice.

**Figure 1.**
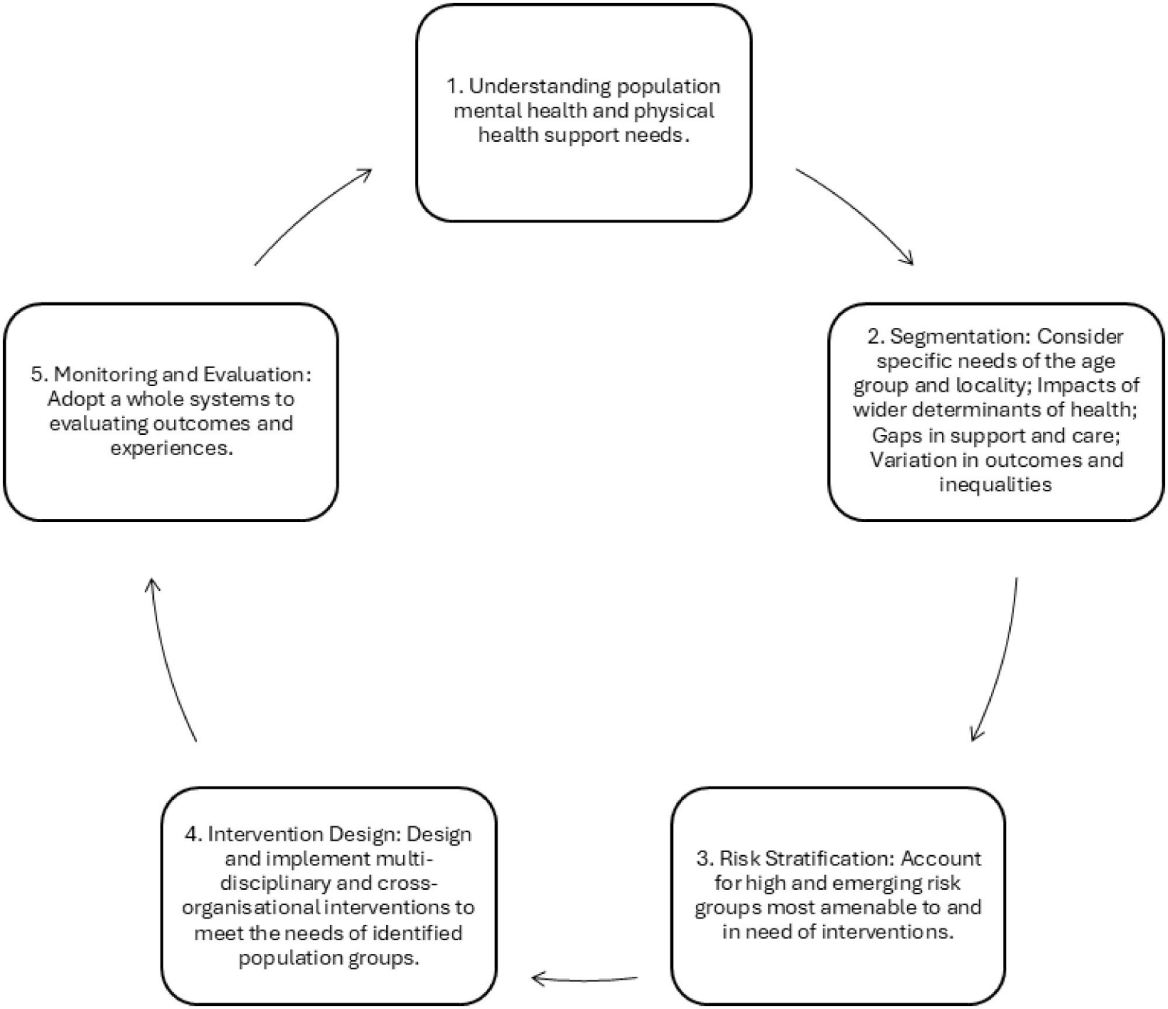
Intelligence led public health.

The urgency of data sharing during the COVID-19 pandemic demonstrated the possibilities of collecting and sharing data rapidly and securely, and that these data flows—between organizations, both national to local and local to national—can increase our ability to understand upstream influences on health, as well as design and test interventions [2, 3]. PHM has been used to identify inequalities, segment populations (e.g. locally or sub-population), and stratify risk, supporting targeted interventions (see Fig. 1).

Examples include PHM to improve access to children’s mental health services [4], identify potential domestic violence victims before police involvement [5], and link housing and health data to address respiratory and cardiovascular conditions [6]. NHS England outlines core PHM capabilities as robust data governance, analytical capacity, and alignment between identified needs and targeted interventions [2].

However, PHM faces technical and organizational barriers, including incomplete data, lack of linkage between sectors, limited analytical capacity, and regulatory constraints [7, 8]. Main *et al.* [8] also identified mindset-related challenges that include the proactive *versus* the reactive nature of health care which means lower priority is accorded to identifying those at risk of developing conditions, patient mistrust of data sharing, and workload pressures.

The declining mental health of young people is a pressing concern. Globally, one in seven 10- to 19-year olds experience a mental health condition [9] that makes them particularly vulnerable to social exclusion, discrimination, stigma (affecting readiness to seek help), educational difficulties, risk-taking behaviours, physical ill-health, and human rights violations [9]. In England, probable mental health disorders in 7- to 16-year olds rose from 12.1% in 2017 to 16.7% in 2020, yet many young people remain unrecognized and untreated [10]. Local intelligence in the study area indicated worsening mental health and wellbeing indicators, including increased self-harm, suicide, and lower reported happiness among school pupils (through the Schools Health Education Unit survey) [11].

Currently, there is limited evidence on PHM implementation for mental health. The explicit use of PHM for public health programmes remains uncommon, despite emerging mental health informatics hubs such as DATAMIND [12] and the ECHILD project [13]. These initiatives demonstrate the potential of linked datasets, but challenges remain in applying PHM at the local level to inform programme design.

We conducted a multi-phased evaluation of a mental health programme targeting young people aged 16–24 years at risk of mental health conditions in a region in the East of England. The mental health programme was composed of four intervention streams, namely Upskilling our workforce—mental health training delivered to teachers, and community leaders working with young people, Wellbeing navigators—8 weeks of mental health support tailored to provide guidance to young people navigating periods of transition (e.g. moving from education to the workplace), Community collaborations—development of a network of community groups to better link up existing support available, and Building resilience—a whole-school approach to improving mental health in young people. The programme was funded through the regional Integrated Care System (ICS), which had adopted a PHM strategy to inform commissioning decisions and drive integrated service delivery. The PHM strategy aimed to use linked data and collaborative planning to target resources where they would have the greatest impact, particularly in addressing health inequalities in priority areas such as young people’s mental health. The evaluation phase described in this paper focused on understanding stakeholders’ experiences of applying PHM in practice, both its opportunities and its challenges, to inform future commissioning and programme design.

## Methods

### Theoretical underpinning

Following guidance of the Medical Research Council (MRC) [14] this evaluation adopts a programme theory that aims to understand how and under what circumstances programmes lead to change. Not only is the focus on whether the programme is effective in improving the mental health and wellbeing of young people, but also on how it might do this. Theory-based evaluation relies on a programme theory explaining how an intervention is expected to generate its intended outcomes. Creating a logic model [15] is an initial stage of evaluation which helps to map out the underlying assumptions and theories of change on the factors that drive its effectiveness This took place through three coproduction workshops facilitated by the evaluation team with participation from local stakeholders from public health, mental health services, a college of further education (*n = *10), and a public contributor [Public and Patient Involvement and Engagement (PPIE) representative] to formulate these short-, medium- and longer-term outcomes.

**Figure 2.**
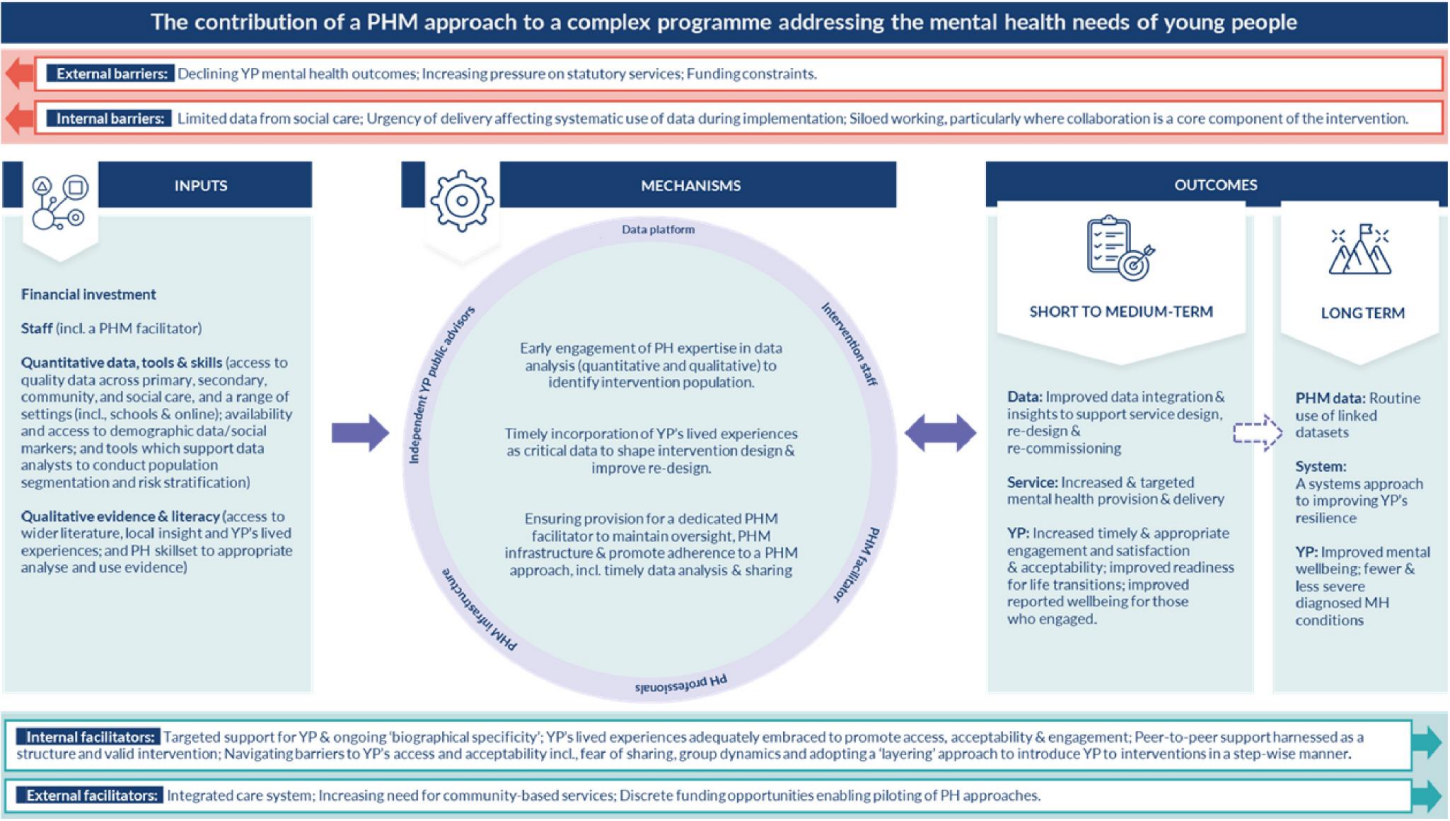
Logic model demonstrating the contribution of a population health management approach to mental health programme design and implementation.

Figure 2 summarizes the activities, resources, knowledge, and skills required to design and deliver the programme. The mechanisms that helped steer the programme towards desired outcomes are shown highlighting the importance of data science expertise but also local qualitative insights.

During the co-production, participants identified various assumptions and expected outcomes from the PHM approach which are summarized in Table 1 and were used to design the wider evaluation of the mental health intervention.

**Table 1.** Theory of change illustrating the rationale and assumptions underpinning a population health management approach to a complex mental health intervention for young people.

	If statement	Then statement
1	IF existing local linked datasets and intelligence is (a PHM approach) are used alongside qualitative data…	THEN it will be possible to identify the mental health needs and target populations amongst young people
2	IF a programme of interventions is designed based on a PHM approach…	THEN service provision will grow, and the workforce will be better able to respond to the needs of young people
3	IF a programme of interventions is designed based on a PHM approach…	THEN young people will engage with services earlier, find services more acceptable, and have higher resilience.
4	IF young people engage with services earlier, find services more acceptable and have higher resilience…	THEN they will be more ready for life transitions, report improved well-being, reduced levels of self-harm and experience reduced levels of stigma.
5	IF a PHM approach is successfully applied…	THEN linked data sets will be routinely used for assessing need and managing service provision.

### The evaluation design and analysis

**Figure 3.**
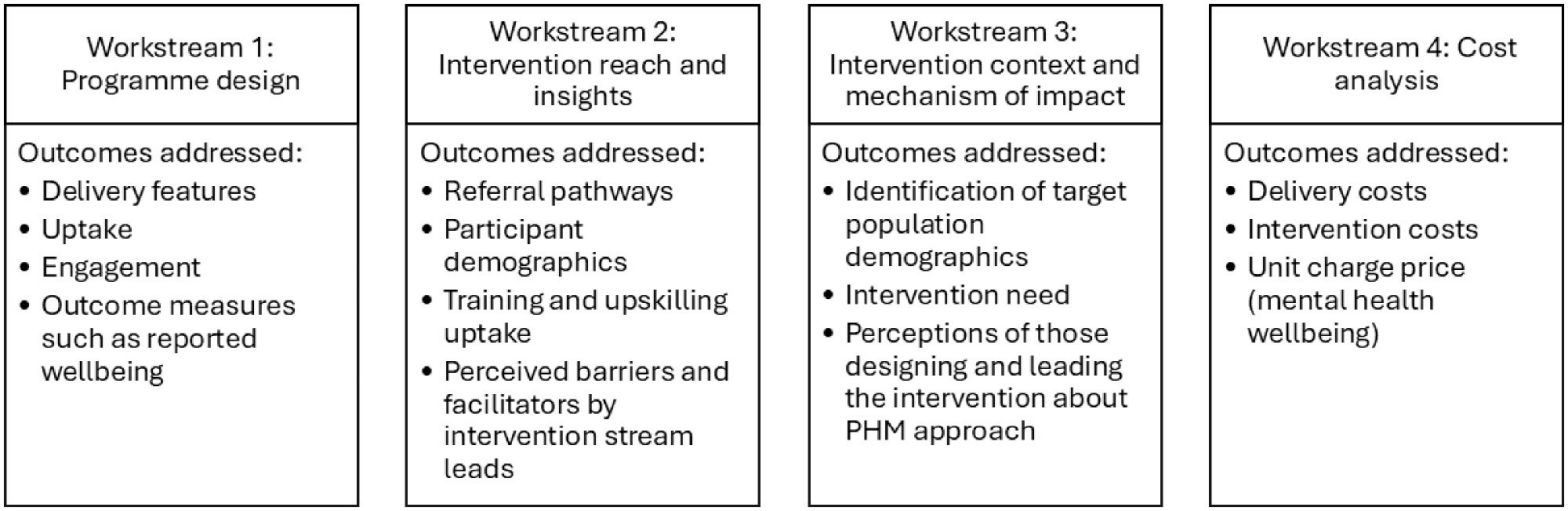
Study design and evaluation components for the monitoring phase of the conducted evaluation. The four workstreams and the outcomes addressed are aligned to the key components of a process evaluation according to the MRC’s framework for developing and evaluating complex interventions.

The evaluation of the mental health intervention utilized a parallel mixed-methods, theory-based design, with four work packages as shown in Fig. 3. The aim of the evaluation was to assess the effectiveness of a mental health intervention for 16–25 years olds identified as most in need of support.

This paper reports on findings from work package 3 which included in-depth qualitative semi-structured interviews with public health professionals (including mental health practitioners, public health officers in the council, and a public health registrar) (*n = *5), intervention leads and two community hub leads (*n = *3), and a focus group with young people who had contributed to interventions design (*n = *5). Participants were asked about the role of PHM in needs assessment and intervention design, perceived benefits and challenges of the PHM approach, and the nature and usefulness of the data employed. Data from the focus group and interviews were inductively and deductively analysed and synthesized into themes using Delve.io software.

### Patient and public involvement

The evaluation team were supported by a patient and public engagement (PPIE) panel also known as Public Contributors made up of five young people who lived in the locality of the programme setting. They supported the design of research tools and advised on the evaluation process and how the programme was being implemented.

### Ethical approval

The study was carried out in accordance with the Declaration of Helsinki and received ethical approval from the School of Health and Social Care Ethics Panel ETH2223-0200 and ETH2324-0074. All participants gave written consent for their data to be used.

## Results

The views of the participants are presented here with selected verbatim illustrative quotes in relation to their experience of the role PHM in needs assessment and programme design, the perceived benefits and challenges of the PHM approach including the infrastructure needed, and the nature and usefulness of the data employed. Public health professionals describe the PHM approach:

- Providing more integrated and coherent linking of data to provide a picture of mental health.
- Directing the planning for redesign of services and delivery to better meet the needs of young people as identified from improved data insights.
- Stimulating particularly strong integration of data within and across data types (quantitative and qualitative) during intervention design, including:
- Use of linked quantitative datasets (integration within data types);
- Incorporation of qualitative data from wider literature, local insight, and young people’s lived experience (integration across data types).

### Understanding and experience of PHM approach

Participants understood PHM as an approach that integrates multiple data sources to provide a more complete picture of population health needs. In this programme, quantitative datasets (e.g. primary care, acute care, 111 calls, ambulance and mental health trust data) were linked through a population analytics platform, with a specialist data scientist leading the analysis. An aim of a PHM approach is to provide a better picture of health needs using multiple data sources.

‘So we [used] a segmentation tool… We also [used] primary care, acute, 111, ambulance and mental health trust data. And this is available as a linked dataset within our population analytics platform, which has been made available to us’.—PH professional 3

We found evidence of some integration and combining of data: this was particularly strong within quantitative datasets, and across qualitative and quantitative datasets. As one PH professional put it:

‘I think we had a really good mix, actually, of insights from partners, evidence that was out there, some data and then, actually some lived experience and kind of co-production as part of that as well’.—PH professional 1

This integration helped to inform service design and help to address variation in outcomes (e.g. reported wellbeing) between different groups, including those with protected characteristics and who have difficulty accessing support. However, social markers, including sexual orientation and caring responsibilities, were often absent or inconsistently recorded, limiting the ability to identify some at-risk groups through routine data alone.

‘And I think the social care bit is missing still. But then, what I tried to do is bring it in separate[ly] and try to triangulate to tell the story. But it’s not linked to get the social care. But, yes, within our patch, we have got primary, secondary, and tertiary care and community health services data in one place… I had to analyse children’s vulnerable data separately. It would have been nice if there was a code, and I could link and see…’.—PH professional 3

‘Social markers were difficult, first of all some of it just wasn’t available particularly the more social parts so if a young person hadn’t attended a GP you wouldn’t have that data but also even if they had, it wouldn’t necessarily be in such a way that it would flag up on the system’.—PH professional 1

Engagement with PHM principles varied. Some stakeholders saw it as adding rigour and detail to their usual data-led planning, and as an opportunity for more integrated and coherent linking of data to provide a picture of mental health; others described it as ‘new wine in an old bottle’, seeing the approach as similar to their usual ways of working.

### Infrastructure and facilitation supporting the PHM approach

The availability of a dedicated PHM facilitator and data scientist was widely viewed as critical to the programme’s ability to analyse and interpret linked datasets. This infrastructure enabled more detailed understanding of needs, including patterns that would not normally be visible in routine reporting. However, some participants noted that the datasets reflected a narrow, clinical view of mental health, capturing self-harm, overdoses, and alcohol intoxication, while missing less acute but significant wellbeing concerns. Public health professionals were aware that many of the mental health issues faced by young people would not ‘flag’ in data sources:

‘Thinking of PHM as an approach where you could get data and then look at going to an individual level and think right, we want to go to GPs and ask them to contact these people who we think might be at risk and haven’t sought help. Actually, that was going to be a bit more tricky because thinking about the people who came up on our system where we do have huge risk factors that wouldn’t necessarily be registered in a GP surgery’.—PH professional 1

Gaps persisted, particularly in linking health and social care data. Attempts to connect datasets for young carers, looked-after children, and other vulnerable groups on a single platform were often unsuccessful due to system incompatibility or governance barriers.

‘So that [linking data across social and health care] would have been really quite useful, if we could have linked the young carers that we had from local authority with GP and then maybe hospital data that could have been really useful, but as far as I’m aware we couldn’t do that, and I know that [name] requested … I know [name] was trying and finding it quite hard to get the data for looked after children [at one point]’.—PH professional

The systematic segmentation and cohort analysis enabled by PHM tools provided a more granular understanding of patterns by practice, age, ethnicity, and prescribing data than was previously available, but did not replace the need for local insight.

‘I think there’s more of an emphasis on data, on cohorts, on segmentation but otherwise I don’t think it’s fundamentally different from the way that we approach most of what we do. So, in that way, my prior experience has always been about using data, trying to identify where outcomes are poorer, whether that’s an age cohort, a geographical cohort, a care group cohort, so you are always using data to look where there’s anomalies or poorer outcomes. What I think is different here is that with the help of the Commissioning Support Unit, I don’t think I ever had data, a spreadsheet, that showed me by practice, age, cohort and things like what were the ethnicity, what were the people being prescribed, what their age was, I don’t think I’d ever had such a detailed understanding of where those issues might be but also an understanding of the gaps in the data as well. It felt like it was doing what we would do automatically but doing some elements with much more rigour and detail.’—PH professional 2

Local qualitative intelligence, and incorporation of the lived experience of young people participating in the interventions, was essential for understanding social and environmental determinants.

‘We could look at the recorded rates of mental ill-health in 16- and 17-year olds and it was really, really low and then suddenly when they became 18, 19, the reporting of that data went way … you know, it jumped up and I don’t think you suddenly develop a mental health condition at the age of 18, I think before then there was a reluctance to have that recorded as a diagnosis on the GP record or seek help independent of your parent… ‘Intervention Lead 2

### Planning for redesign of services and delivery

The integration and linkage of data insights informed service and intervention design but the identification of groups as most in need such as young people in care or who have left care, are LGBTQ+, and those who are neurodiverse was through other methods. For example, one of the three arts-focussed ‘community hubs’ that was developed to provide support within the community and outside of the school environment, provided targeted support for young carers and was developed based on literature and case reports:

‘We settled on trying to target young people who are LGBTQ+ or questioning, and that came out from some of the focus groups we’d done with young people. Knowing that young carers and care leavers suffered with mental health difficulties was something we found in wider literature and also from case reviews from local authority data’.—PH professional 1

## Discussion and conclusion

A PHM strategy aims to make better use of data to improve understanding of patterns of poor health and wellbeing, identifying those at higher risk of poor outcomes based on bio-psycho-social risk factors and thus helping to tailor interventions according to their ‘impactability’. This study examined the application of a PHM approach to designing a mental health intervention for young people, providing an opportunity to explore how PHM principles operate and are perceived within a public health context.

### Interpreting the findings in the context of PHM

PHM is intended to move services from reactive to proactive, tailoring interventions based on risk profiles and social determinants. The approach is an asset for tackling population-level health priorities, so too is its focus on reducing inequalities in its delivery of health and care interventions. It has the potential to support upstream prevention of multiple burden of disease; promote collaboration across health, social care, public service, and voluntary sectors; and shift the mode of care from reactive to proactive and preventative [16].

When evaluating the mental health programme described in this paper, the PHM approach offered more granularity and rigour than some stakeholders had previously experienced, for example as a result of integration within data types (e.g. linked quantitative datasets) and across data types (e.g. combining routine data with qualitative local insights and young people’s lived experiences), particularly with support from a dedicated data scientist and PHM facilitator.

Yet, the findings suggest that challenges remain. Despite the intervention having a dedicated specialist data scientist, technical limitations and workforce skills gaps were evident (e.g. data analytics). This has been highlighted in previous research (e.g. see [1, 8]). What is more, there are challenges in data access, and linkage. Access to data and data sharing was reported as much easier within the NHS than in local government where linking demographic data such as Index of Multiple Deprivation (IMD) and social care data with health outcomes proved difficult. As a result of the challenges around data, PHM in public health cannot rely solely on linked administrative datasets. In this evaluation, this was recognized as an issue, and target groups were frequently identified through qualitative intelligence and stakeholder input rather than ‘big data’ alone.

The current approach to building a picture of mental health needs in a population presents with limitations, primarily the over-reliance on viewing young people’s mental health through a medical or diagnostic lens. Labels such as depression and anxiety for young people’s mental health challenges, together with use of prescribing and hospital admissions data, are examples. Additional data limitations include restriction of population-level health surveys to the school environment, hence potentially failing to include adolescents not in school, and who may face significant health inequalities [17]. The Warwick Edinburgh Mental Wellbeing Scale (WEMWBS) was also used in this study to assess wellbeing: however, it was not originally designed for use with young people, although some studies do report that it is validated for this age group [18].

### Implications for practice and policy

Our evaluation highlighted the positive opportunity afforded to a PHM approach, when quantitative data are combined with young people’s lived experiences to shape intervention design. However, our evaluation raises questions about the scope and nuance of PHM when applied to complex, socially mediated outcomes such as mental health.

PHM is not a term that is widely or similarly used. Although it is clearly outlined in various documents [2], it is used differently according to its purpose and context [19, 20]. This was evident in this research as participants did not share a common understanding of the approach. As a result, they shared experiences of employing PHM in design and implementation of a public health programme, rather than pinpointing the precise mechanism of operation.

Moreover, PHM’s segmentation and targeting must be balanced with proportionate universalism [21], ensuring universal offers (e.g. community hubs) are adapted in intensity and form for different subgroups. Moreover, investment is needed in capturing relevant social determinants within datasets, particularly for groups whose needs are under-recorded. Dedicated analytical capacity and facilitation are important enablers of PHM, but many local public health teams face skills gaps and fragmented systems [8]. Finally, effective PHM depends on governance arrangements that enable cross-sector data sharing, particularly between NHS and local authority systems.

### Contribution of this study

This study adds to the limited literature on PHM in public health, especially in relation to young people’s mental health. It provides empirical insight into both the opportunities and constraints of operationalizing PHM in a local system with an explicit PHM strategy. While the systematic data analysis provided by PHM tools was valued, stakeholders emphasized that it complemented rather than replaced local, relational, and experiential knowledge. Local governments should ensure that these and wider evidence as well as young people’s views are included in decision-making when identifying those most in need of mental health support and how best to support them.

Key points

- Population health management is an emerging approach that uses available data to identify populations at risk.
- Mental health needs are not easily, or fully identified by available data and there are challenges in linking data across sectors.
- Specialist skills, such as data analysis, are needed for effective population health management.
- Population health management is not a magic bullet, and a range of types of data, including qualitative insights, are needed to be identify mental health needs.

Conflict of interest: The research was conducted in the absence of any financial or commercial relationships that could be construed as a conflict of interest.

## Data Availability

Anonymized data is available from the corresponding author upon reasonable request.
